# A Moderated Mediation Model of the Influence of Cynical Distrust, Medical Mistrust, and Anger on Vaccination Hesitancy in Nursing Staff

**DOI:** 10.3390/ejihpe13110167

**Published:** 2023-10-29

**Authors:** Athanasios Tselebis, Christos Sikaras, Charalampos Milionis, Eleni Paraskevi Sideri, Konstantinos Fytsilis, Styliani Maria Papageorgiou, Ioannis Ilias, Argyro Pachi

**Affiliations:** 1 Psychiatric Department, Sotiria Thoracic Diseases Hospital of Athens, 11527 Athens, Greece; kosfits@gmail.com (K.F.); stellamar4@yahoo.gr (S.M.P.); irapah67@otenet.gr (A.P.); 2 Nursing Department, Sotiria Thoracic Diseases Hospital of Athens, 11527 Athens, Greece; cris.sikaras@gmail.com; 3 Department of Endocrinology, Diabetes and Metabolism, Elena Venizelou General and Maternity Hospital, 11521 Athens, Greece; pesscharis@hotmail.com (C.M.); iiliasmd@yahoo.com (I.I.); 4 Emergency Department of General Hospital of Athens Korgialeneio—Benakeio Hellenic Red Cross, 11526 Athens, Greece; siderhlena@gmail.com

**Keywords:** vaccination hesitancy, medical mistrust, cynical distrust, anger, nurses, COVID-19 pandemic

## Abstract

During the pandemic, nurses experienced anger that stemmed from a sense of threat, frustration, or even a sense of injustice. The purpose of this study was to examine the relationship between vaccination hesitancy, anger, cynicism, and medical mistrust among nurses, as there are no relevant studies in the literature. This study was conducted online by completing self-report questionnaires. The Dimensions of Anger Reactions-5, the 8-item “Cynical Distrust” scale, and the Medical Mistrust Multiformat Scale were used. For vaccination hesitancy, two questions with a 5-point scale were used: one question examining hesitancy to get vaccinated with the COVID-19 vaccine, and another question examining hesitancy to get vaccinated with the influenza vaccine. In total, 387 nurses (66 men and 321 women) participated in this study. Nurses showed statistically greater hesitancy toward the COVID-19 vaccine compared to hesitancy toward the influenza vaccine. The variation in vaccine hesitancy was explained by the scores in the Medical Mistrust Multiformat Scale, the Dimensions of Anger Reactions, and the Cynical Distrust Scale. The Medical Mistrust Multiformat Scale mediated the relationship between the Cynical Distrust Scale and total vaccine hesitancy. The Dimensions of Anger Reactions Scale significantly moderated the indirect effect of the Cynical Distrust Scale on total vaccine hesitancy through the Medical Mistrust Multiformat Scale. In conclusion, it is highly likely that anger is involved in reported vaccine hesitancy both by activating schemas of distrust in others and by adopting anti-systemic views of mistrust in the medical system.

## 1. Introduction

The coronavirus pandemic took health systems by surprise and caused a global health crisis with a huge physical and psychological impact [1,2,3]. On a worldwide scale, as well as in Greece, particularly in the first year of the pandemic, the shortages in protective equipment [4] and the containment measures taken by governments [5] contributed to the increased anger of health professionals [6], who also experienced the greatest anxiety and stress [7,8]. 

On the other hand, in the first 18 months of the pandemic, there are reports that over 80,000 health workers worldwide died from severe acute respiratory syndrome coronavirus 2 (SARS-CoV-2) [9]. The increased risk for healthcare professionals is certainly due to close and long-term contact with Coronavirus Disease 2019 (COVID-19) patients and occupational exposure to the virus [10]; meta-analyses report that over 51% of healthcare workers were infected in the first year [11], and 0.5% died [12]. 

Health workers were among the first groups to be offered COVID-19 vaccines worldwide [13] early in the second year of the pandemic. However, a major problem that had to be overcome was the reluctance to get vaccinated, both in the general population [13] and among health workers.

Vaccine hesitancy is defined by the World Health Organization as “delay in accepting or refusing vaccines despite availability.” It is further described as “complex and context specific, varying across time, place and vaccines; influenced by factors such as complacency, convenience and confidence” (namely, the “Three Cs” model of vaccine hesitancy) [14]. Among these factors, it is probably less likely that complacency and convenience would have influenced the behavioral decisions of health workers to accept, delay, or reject COVID-19 vaccines, which were conveniently offered at their disposal, because they have witnessed the COVID-19-associated morbidity and mortality. However, other factors, such as confidence and trust, are probably among the most prominent determinants of vaccine hesitancy. It should be emphasized that vaccine hesitancy among health workers predates COVID-19 vaccines. Those health workers who were hesitant to vaccinate before the pandemic expressed objections related to vaccine safety, violation of personal autonomy, and mistrust in government authorities [15,16].

Recently, the term ‘vaccine hesitancy’ has been redefined as “a psychological state of indecisiveness regarding a vaccination decision” in order to separate the term from vaccination behavior [17]. Vaccine hesitancy exists along a continuum from total vaccine refusal to mild concern or skepticism, implying varying degrees of indecision [18]. This definition emphasizes the dynamic nature of vaccine hesitancy, as it fluctuates over time in response to diverse influencing factors. Accordingly, the model explaining vaccination hesitancy has been updated to include complacency, confidence, constraints, calculation, and collective responsibility as the 5C scale to monitor psychological antecedents of vaccination [19]. 

In this sense, and in the search for the underlying constructs of anti-vaccination attitudes, relevant individual psychological barriers, such as cynicism, influencing the decision-making process regarding vaccination intention deserve to be studied, especially among nurses who have been caring for COVID-19 patients and significantly contribute to the vaccination of the public against COVID-19 [20]. Cynicism is an attitude characterized by a general distrust of the motives of others (in the present study, it is defined as an individual’s more general distrust of others [21]).

Concerns about the safety and efficacy of the COVID-19 vaccines, misinformation and lack of knowledge, and distrust in the vaccine development process, pharmaceutical companies, experts, and government agencies among healthcare workers have already been studied [22]. Conceptually different from distrust, mistrust refers to an ongoing monitoring process of credibility, reflecting caution and skepticism [23]. Medical mistrust is the extent to which people do not trust the healthcare system, government health services, medical scientists, and doctors or other health professionals [24]. It has been argued that this mistrust may partly be due to a lack of institutional support during the pandemic [25]. In fact, there is a paucity of studies in nursing staff that assess mistrust in the healthcare system and that examine its relationship with vaccination hesitancy. 

Another factor that has not been adequately explored in terms of vaccine hesitancy in nurses is anger. Previous studies support that negative emotions, specifically fear and anger, generate vaccine hesitancy [26]. Anger is one of the basic human emotions, involving a complex set of physiological and psychological responses to feelings of threat, frustration, or even a sense of injustice [14,27]. It is characterized by feelings of resentment, hostility, and arousal, as well as cognitive appraisals that trigger an impulse to an aggressive or assertive response [14,28,29]. 

The majority of frontline healthcare staff having a prominent role in the proper functioning of healthcare systems and providing specialized services to patients are nursing personnel [9]. At the same time, they are the professional group that became the most popular during the pandemic crisis to the point of recognizing the nursing profession as heroic. Yet, it is a professional group that showed increased vaccination hesitancy [30]. It is therefore necessary to examine the factors associated with this reluctance in order to interpret and address it. 

The World Health Organization declared an end to the COVID-19 pandemic on 5 May 2023, perceiving an increase in immunity from vaccination and infection leading to fewer deaths. In Greece, the suspension of unvaccinated health workers extended until the end of the year 2022. The timing of this study is justified by our choice to conduct the study after the end of the pandemic a few months after the unvaccinated personnel had returned back to work to measure the steady state of nurses’ vaccine beliefs and intentions after the extreme negative emotions that could blur their cognitions had subsided. 

In the present study, we aimed to explore the relationships among vaccination hesitancy, trust in the healthcare system, cynical distrust, and anger in nursing staff and to test a moderated mediation model of the influence of cynical distrust on vaccination hesitancy mediated by medical mistrust and moderated by anger. The proposed hypotheses were as follows:

**Hypothesis** **1:** *Cynical distrust would be positively related to vaccination hesitancy*.

**Hypothesis** **2:** *Medical mistrust would mediate the effect of cynical distrust on vaccination hesitancy*.

**Hypothesis** **3:** *Anger would moderate the indirect relationships between cynical distrust and vaccination hesitancy via medical mistrust*.

## 2. Subjects and Methods

### 2.1. Research Design and Procedure

This was a cross-sectional study. The target group of this study consisted of all registered currently employed Greek nurses. Self-report questionnaires were used to collect data. These questionnaires were distributed by the authors via email. The invitation via email contained an anonymous link that allowed access to the online survey platform. On the first page of the online questionnaire, we assured potential participants that (a) participation in the survey was voluntary and (b) a statement of consent was given. The participants’ email addresses were retrieved through links to the websites of Greek nurses from their scientific and professional societies. The study sample included nursing staff who agreed to respond to the email as a convenience sample, and no measures were taken to increase the response rate apart from the reassurance of data privacy. This study was conducted from 1 June to 20 June 2023. With a target population of 27,103 nurses [4], at a 95% confidence level, 5% margin of error, and a 50% confidence rate, an adequate participating nurses’ sample size was set at 379 participants; to this end, 500 invitations were sent via email, and 387 nurses accepted to respond (response rate 77.4%).

### 2.2. Ethical Considerations

In the invitation, we informed the nurses about the purpose and design of our study. In addition, we ensured that they gave electronic informed consent by asking: “Do you agree to participate in this study?” as the first question in the online questionnaire. Only nurses with a positive response were allowed to continue in completing the questionnaire. This study was conducted in accordance with the ethical principles as defined by the Declaration of Helsinki, the International Committee of Medical Journal Editors, and the General Data Protection Regulation (GDPR-2016/679) of the European Union. This study has been approved by the Clinical Research Ethics Committee of “Sotiria” General Hospital (Number 20649/23).

### 2.3. Measurement Tools

Initially, the participants were asked to indicate their gender, age, and years of work experience. Then, they completed the following questionnaires.

#### 2.3.1. Dimensions of Anger Reactions-5 (DAR-5)

The DAR-5 scale is a short 5-item scale that measures the experience of anger. Respondents rate their experience of anger on a 5-point Likert scale ranging from 1 = never or almost never to 5 = always or almost always. The 5 scores are summed, with a total score ranging from 5 to 25. Low scores indicate a low experience of anger, while higher scores indicate a more severe experience of anger. The cut-off point for the scale is ≥12 [6,31,32,33]. Regarding the internal reliability of the questionnaire in the present study, Cronbach’s alpha was equal to 0.811.

#### 2.3.2. Eight-Item “Cynical Distrust” Scale (CDS)

The Cynical Distrust Scale (CDS) [21] was used to measure cynicism. The scale includes 8 items [34,35], which, in the Greek version, are rated on a 5-point Likert scale ranging from 1 = strongly disagree to 5 = strongly agree [36]. A representative question of CDS is: “It is safer to trust nobody?” The 8 scores are summed, with a total score ranging from 8 to 40. A high sum in the score of the 8 items indicates a high value of cynical distrust. A value of 25.5 is an indicative average value in the Greek general population [36]. The internal reliability of the questionnaire in this study was very good, with Cronbach’s alpha at 0.814.

#### 2.3.3. Vaccine Hesitancy

In most studies, the strength of hesitancy to receive a vaccine is recorded by asking a relevant question. In the present study, we used two questions to capture vaccination hesitancy. In the first question, we stated: “If a new booster dose was recommended and available to you as an adjunct to the current COVID-19 vaccination schedule, would you get vaccinated?” In the second question, we asked: “In the next recommendation for the influenza vaccine, would you get vaccinated?” In both questions, the respondent had to choose a response on a Likert scale of 1: Definitely yes, 2: Probably yes, 3: Not sure, 4: Probably no, 5: Definitely no. We used the sum of the two questions to obtain an overall vaccine hesitancy score that took values from 2 (no hesitancy at all) to 10 (high hesitancy). The internal reliability of the two questions had a Cronbach’s alpha of 0.724.

#### 2.3.4. Medical Mistrust Multiformat Scale (MMMS) 

The scale includes 6 items. Its main feature is that it uses multiform response options for each item. Thus, the item “Do you feel that the medical authorities are trustworthy and honest?” can be answered on a scale from 1 to 5 with options ranging from “I feel exactly like that” to “I definitely don’t feel like that.” Meanwhile, the item “How much would you agree that medical authorities are more interested in making money than taking care of people?” can be answered on a scale of 1 to 7, with options ranging from “strongly agree” to “strongly disagree” [24]. The internal reliability of the questionnaire in this study was very good, with Cronbach’s alpha of 0.832.

### 2.4. Statistical Analysis

Initially, the data were analyzed using descriptive statistics so that continuous variables were expressed as means and standard deviations. We used the x^2^ test to assess gender differences and the two-sample *t*-test to test whether the mean age and years of work experience in this sample agreed with findings from other recent studies on Greek nursing staff. The means of continuous variables as to gender were tested with the independent samples *t*-test. The effect size was calculated with Hedges’ g-value from the results of the independent samples *t*-test, considering that values less than 0.2 suggest a small effect size, values less than 0.5 suggest a medium effect size, and values less than 0.8 suggest a large effect. We used Pearson’s correlation to test the direction and strength of correlations between continuous variables. Linear regression models were constructed to investigate whether the associated variables were significant predictors of the dependent variable of total vaccine hesitancy. Assumption testing for the regression analysis was conducted. Simple Mediation and Moderated Mediation analyses were performed using the Hayes SPSS Process Macro v4.0 [37,38,39], models 4 and 7. Significant effects are supported by the absence of zero within the confidence intervals. Statistical significance was set at *p* < 0.05 (two-tailed).

## 3. Results

This study involved 66 men and 321 women. In this study, the sex ratio was not statistically different (x^2^ *p* > 0.05) from the sex ratio in a recent study of Greek nurses [7]. There was no difference in either mean age (43.46 years) or mean years of work experience (18.03 years) in the present study compared to the means in relevant recent studies (42.88 age and 17.96 years of work experience, two-sample *t*-test *p* > 0.05) [7]. Slightly more than one third (35.4%) of the responders showed anger values greater than 12. Cynicism in nurses (24.55 ± 6.23) showed a lower mean value (sample *t*-test *p* > 0.05) compared to the value of 25.5 obtained in the past in the general population [36].

The descriptive characteristics of the participants are presented in Table 1. Women showed higher scores on the Cynical Distrust Scale (*t*-test *p* < 0.01, 24.96 ± 6.10 vs. 22.58 ± 6.53, Hedges’ g: 0.39) and Dimensions of Anger Reactions Scale (*t*-test *p* < 0.05, 11.48 ± 3.96 vs. 10.35 ± 3.18, Hedges’ g: 0.29) compared to men. No gender differences were found in vaccination hesitancy (*t*-test *p* > 0.05).

The nurses’ expressed reluctance to uptake the COVID-19 vaccine is statistically greater (Table 1) than the reluctance for the influenza vaccine (paired *t*-test *p* < 0.01 3.58 ± 1.33 vs. 2.68 ± 1.49, Hedges’ g: 0.63). Table 2 shows the frequencies and percentages of responses to the questions on vaccine hesitancy. In total, 32.3% of the nurses expressed willingness and intention to get vaccinated with the influenza vaccine, whereas the rest were hesitant and, among them, 15.2% were reluctant and 17.3% unwilling. On the other hand, for an additional dose of the COVID-19 vaccine, only 9.8% of nurses expressed willingness and intention to get vaccinated, whereas the rest were hesitant and, among them, 23.8% were reluctant and 33.3% refused.

Strong positive correlations were evidenced between vaccine hesitancy, anger, cynicism, and medical mistrust (Pearson Correlations *p* < 0.01, Table 3). Age showed a statistically high negative correlation with both the cynicism scale and the medical mistrust scale (Pearson Correlations *p* < 0.01). Work experience showed a negative correlation with both the cynicism scale and the medical mistrust scale (Pearson Correlations *p* < 0.05) and a strong correlation with age (Pearson Correlations *p* < 0.01). The correlation was strongly positive between vaccine hesitancy toward the COVID-19 vaccine and vaccine hesitancy toward the H_1_N_1_ vaccine (Pearson Correlations *p* < 0.01).

Before conducting regression analyses, we examined whether the necessary assumptions were met. The absence of multicollinearity in the data was assessed by implementing Variance Inflation Factor analysis (VIF), with values ranging from 1.37 to 1.27 (Table 4). The independence of the residuals was checked with the Durbin–Watson test, with a value of 1.957 (Table 4). Normality was checked through visual inspection of the predicted probability (P-P) plots. Homoscedasticity was examined through visual inspection of the scatter plot of regression standardized residuals and regression standardized predicted values.

Using the Stepwise method, we performed a multiple regression analysis to identify which factors best explained the total vaccine hesitancy scores. In the multiple regression analysis, we defined total vaccine hesitancy as the dependent variable and as the independent variables age, work experience, Cynical Distrust Scale scores, Dimensions of Anger Reactions scores, and Medical Mistrust Multiformat Scale scores. Multiple regression analysis showed that the Medical Mistrust Multiformat Scale scores explained 5.8% of the variance in the dependent variable, while a further 2.2% was explained by the Dimensions of Anger Reactions variable, and 1% was explained by the Cynical Distrust Scale. The other variables did not explain the variance in total vaccine hesitancy (Table 4). 

**Figure 1:**
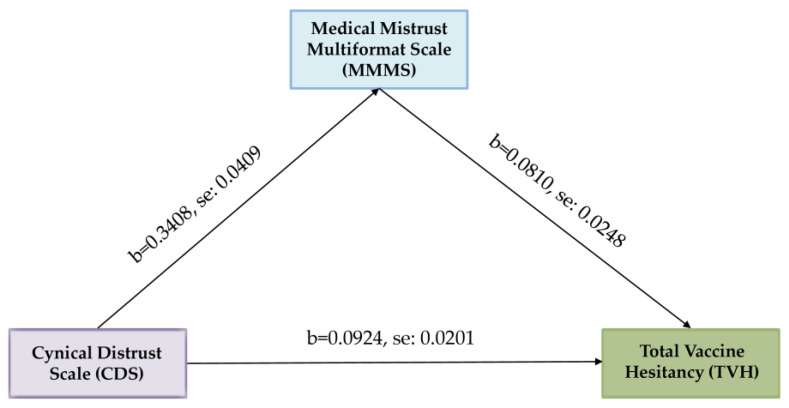
Mediation analysis of Medical Mistrust Multiformat Scale (MMMS) on Cynical Distrust Scale (CDS)—total vaccine hesitancy (TVH) relationship.

To clarify the nature of the relationship between cynical distrust and total vaccine hesitancy, and to answer one of the hypothetical research questions, we investigated the underlying mechanism by which cynical distrust influences total vaccine hesitancy through medical mistrust by performing a simple mediation analysis. The outcome variable for the analysis was total vaccine hesitancy. The predictor variable for the analysis was the Cynical Distrust Scale. The mediator variable for the analysis was the Medical Mistrust Multiformat Scale. Age and work experience were added as covariates. Bootstrapping was performed with the Hayes SPSS Process Macro to examine whether medical mistrust mediated the relationship between cynical distrust and total vaccine hesitancy. Based on 5000 bootstrap samples, the results revealed a significant indirect effect of cynical distrust on total vaccine hesitancy through medical mistrust (b = 0.0276, t = 2.9677). Furthermore, the direct effect of cynical distrust on total vaccine hesitancy in the presence of the mediator medical mistrust was also found to be significant (b = 0.0648, *p* = 0.0029). Hence, medical mistrust partially mediated the relationship between cynical distrust and total vaccine hesitancy, and the model explains 29.87% of the variance in the outcome variable total vaccine hesitancy (Table 5). Age and work experience, as covariates, had no statistically significant relationship. Unstandardized coefficients for the variables with standard errors are illustrated in Figure 1. 

Furthermore, we examined if anger as a moderator is changing the strength of the indirect effect of the above mediation. The index of moderated mediation was significant, b = −0.0025, 95% percentile CI [−0.0051, −0.0006], providing evidence for a moderated mediation. The conditional indirect effect for small values (−1 SD) of the moderator was the strongest, b = 0.0280, 95% percentile CI [0.0101, 0.0497], while it was weaker but still significant for medium values of the moderator, b = 0.0182, CI [0.0064, 0.0322], and for high values (+1 SD) of the moderator, b = 0.0084, CI [−0.0017, 0.0210], it was insignificant.

**Figure 2:**
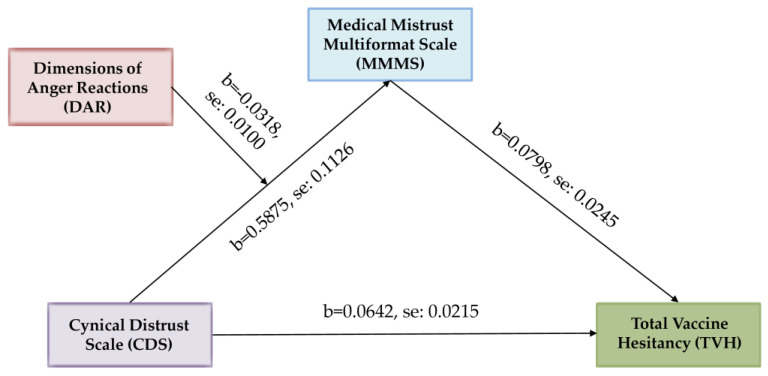
Moderated mediation analysis of the effect of Dimensions of Anger Reactions (DAR) on the association between Cynical Distrust Scale (CDS) and total vaccine hesitancy (TVH) through Medical Mistrust Multiformat Scale (MMMS).

For the path from the independent variable (cynical distrust) to the mediator (medical mistrust), there was a significant interaction between the independent variable and the moderator (Dimensions of Anger Reactions), b = −0.0318, *p* < 0.01, ΔR² = 0.0202. The conditional effect from the independent variable on the mediator was the strongest for small values (−1 SD) of the moderator, b = 0.3511, *p* < 0.001, while it was weaker but still significant for medium values of the moderator, b = 0.2285, *p* < 0.001, and for high values (+1 SD) of the moderator, it was insignificant, b = 0.1058, *p* > 0.05. The path from the mediator to the dependent variable (total vaccine hesitancy) was significant, b = 0.0798, *p* < 0.01. The direct effect from the independent variable to the dependent variable was significant, too, b = 0.0642, *p* < 0.01 (Table 6). Unstandardized coefficients for the variables with standard errors are illustrated in Figure 2.

**Figure 3:**
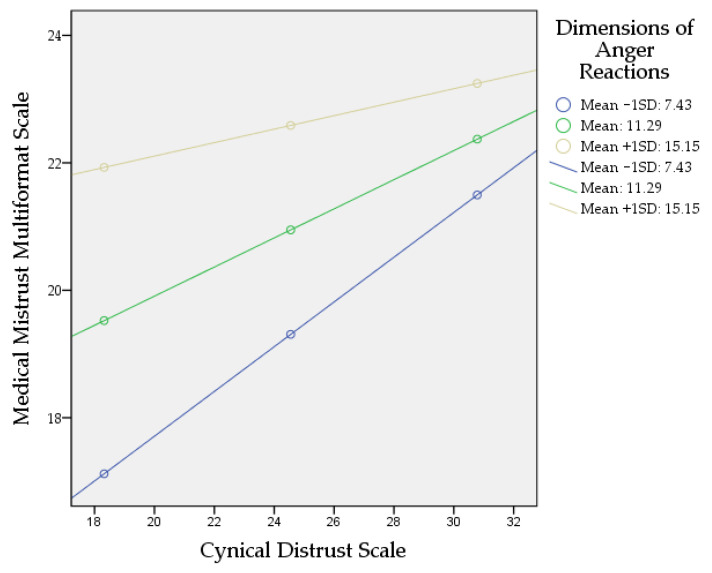
Simple slopes demonstrating the interaction of the moderation analysis.

Figure 3 shows a steeper gradient for low and average Dimensions of Anger Reactions. The impact of the Cynical Distrust Scale (CDS) is much stronger at low and average Dimensions of Anger Reactions. However, at higher Dimensions of Anger Reactions, the line tends to straighten; this shows that at higher Dimensions of Anger Reactions, the increase in the Cynical Distrust Scale does not lead to significant change in the Medical Mistrust Multiformat Scale. According to Johnson–Neyman significance regions at values above 14.6716 of the Dimensions of Anger Reactions, the slope of the Cynical Distrust Scale becomes insignificant *p* > 0.05, meaning that the effect of the Cynical Distrust Scale on the Medical Mistrust Multiformat Scale becomes statistically insignificant.

## 4. Discussion

Several years before the pandemic, the importance of addressing psychological factors associated with vaccine hesitancy was highlighted [16]. During the pandemic, studies confirmed this role of psychological factors [40,41,42,43]. To better understand what leads someone to refuse or delay vaccination, it is important to identify the psychological barriers underlying this decision. In the context of vaccination, previous and recent research show that emotions may influence vaccination intention [44,45,46]. According to vaccine hesitancy research, negative emotions, such as fear, anxiety, and anger, may impact vaccine-related decision making. Among these emotions, anger is more likely implicated against vaccine uptake [18,26,47,48,49], presumably motivating people toward active coping behaviors to maintain or gain a sense of control [50,51]. To our knowledge, this is the first study to investigate the relationship between anger and vaccination hesitancy in nurses. 

In this study, we found the existence of a positive association between anger and vaccination hesitancy. More than one third of the nurses admitted experiencing intense anger emotions post pandemic, whereas a little less than one third expressed such emotions during the first pandemic wave [6]. This difference is more likely attributed to the fact that during the first pandemic wave, nurses were viewed as heroes, but then followed the disillusionment phase. This finding is consistent with the literature, which suggests that increased levels of anger are associated with an increased number of negative life events [6]. We note here that in addition to the negative events experienced during the pandemic, Greek nurses were under particular pressure from the state, with laws making COVID-19 vaccination mandatory for healthcare workers [52]. In Greece, health workers have protested against mandatory vaccination plans. Mandatory vaccination may have paid off, as over 90% of nurses were vaccinated [52]; however, it is possible that this may explain both the anger and the low intention to receive the COVID-19 vaccine booster, as expressed in the sample. Usually, pressure to vaccinate can lower vaccination intention. A study in the UK found that pressure to vaccinate actually increased hesitancy [53]. 

In Greece, acceptance of the influenza vaccine was found to be close to 50% in 2022–2023, with no difference in the rate versus nurses who had the influenza vaccine the previous year [54]. The strong positive relationship between influenza vaccination intention and COVID-19 vaccination intention suggested in the literature [42] was confirmed in the present study; however, it was beyond the scope of this study to search for a causal relationship. That is, the question that needs to be answered in another study is whether hesitancy to vaccinate for COVID-19 will affect vaccination intention in the coming years more generally, or whether it will still affect people’s trust in health systems. 

Another important identified factor influencing vaccine hesitancy already explored among nurses is trust [9]. Research suggests that vaccine rejecters mostly invoke reasons of distrust, display a suspicious or cynical attitude, and hold strong beliefs that others are untrustworthy [55]. They are likely to defy regulations imposed by governments, ignore or resist recommendations or measures taken by policymakers, and are unwilling to follow the advice of scientific experts. Distrusters tend to express vague statements of suspicion or uncertainty, usually without producing conspiracist ideation, but they are eager to endorse any argument against vaccines simply because it is consistent with their attitude [56]. They are usually withdrawn and in a state of disengagement, but, if emotionally charged with anger, they may participate in anti-vaccination groups, often promoting misinformation and challenging vaccination beliefs through empowerment movements [57].

Cynical distrust is associated with a variety of adverse health conditions, either due to biological or behavioral causes [58]. In our study, cynical distrust was found to be statistically lower in nurses than in the general population, which we took for granted, as the nursing profession is characterized by altruism [59]. However, what this study suggests is that cynicism exists in the form of schemas similar to those proposed by cognitive psychology [60], which are activated by negative emotions, such as anger. These schemas will be involved, possibly in a maladaptive way, in the intention or reluctance to get vaccinated. Furthermore, according to the cognitive functional model of the effects of discrete negative emotions, such as anger, elicited through a process of cognitive appraisal of information or misinformation, an integrated decision-making framework is established, predicting subsequent effects on attitudes and behavior [61,62].

Mistrust of the healthcare system, holding negative perceptions of doctors, and negative healthcare experiences, along with the contribution of anger, fuel distrust in vaccine-related professionals and agencies. Instead, positive healthcare experiences and positive views of doctors and medical science are associated with lower vaccine hesitancy. It is worth mentioning that if there is trust in medical science, doctors, or scientific experts, negative emotions, such as anger, do not necessarily impact vaccination decisions [63]. 

In the moderated mediation analysis of our study, anger negatively influences the relationship from cynical distrust to vaccination hesitancy through medical mistrust. Also, the impact of cynical distrust on medical mistrust was much stronger at low and moderate levels of anger, whereas at high levels of anger, its effect became insignificant. A possible explanation according to the theoretical models of anger and persuasion is that anger has the potential to facilitate persuasion; however, more suitable for effective persuasion are lower to moderate levels of anger [62]. High levels of anger render persuasion ineffective because recipients’ ability to engage thoughtfully is likely compromised [64]. This means that cynical distrusters with low or moderate levels of anger are effectively persuaded and likely adopt anti-systemic views of mistrust in the medical system, resulting in vaccine rejection. 

Another important subgroup of hesitators consists of people who are merely reluctant to receive the vaccine, expressing concerns, doubts, and considerations about vaccine safety, side effects, and efficacy or not being a member of high-risk group [55]. Their belief structures based on the weighing of risk and benefit are more amenable to change as opposed to firmly held perspectives stemming from vaccine rejecters. Many of them are adequately informed but yet unconvinced, while others are more receptive to persuasion with potential to re-evaluate their vaccine intentions if provided with sufficiently strong arguments critical for determining the persuasive outcomes. Recent studies have revealed that nurses were more vaccine hesitant compared with other healthcare workers, and this was attributed to vaccine safety concerns [65]. 

Finally, there is uncertainty in the literature as to the role of gender in vaccine hesitancy, with more studies reporting that the male population among healthcare workers is more willing to vaccinate [66,67], while others argue that male respondents were more likely to be anti-vaccine compared to females [68]. No such gender difference was found among the nurses in our sample. 

This study is subject to limitations. First is the use of self-administered questionnaires, which gave a more subjective dimension to the assessment of variables. Second, nurses who do not have easy access to the internet were excluded from the sample, as the invitation was sent through email. Third, gender disproportionality in the nursing samples may have affected generalization to other populations, and the convenience sampling method may possibly reflect selection bias. 

Also, this study investigated limited factors; however, other factors, such as burnout, may be positively associated with both anger [69,70] and vaccination hesitancy [71]. On the other hand, there are factors, such as resilience [71] and social or family support, that are negatively associated with anger [6] and that may reduce vaccination hesitancy [71]. Finally, it should be stressed that vaccine hesitancy is not a fixed attitude but rather changes over time by group and cultural context [72,73].

Overall, it is very likely that anger is involved in the stated refusal of vaccination both by activating patterns of distrust toward others and in the adoption of anti-systemic views of mistrust toward the medical system. We believe that interventions of anger management and/or of enhancing communication skills and stress relief, through educational training programs, for health workers would result in the reduction of anger and anti-systemic thinking. 

## 5. Conclusions

The present study examined the relationships between vaccination intention, anger, cynicism, and distrust of medical authorities. Positive correlations were found between the questionnaires’ scores of vaccine hesitancy, Dimensions of Anger Reactions, the Medical Mistrust Multiformat Scale, and the Cynical Distrust Scale. Although there was a high positive correlation between mistrust and vaccine hesitancy, there were significant differences between the COVID-19 vaccine and the influenza vaccine as to hesitancy rates, with the COVID-19 vaccine showing higher rejection rates compared to the influenza vaccine. Variation in vaccine hesitancy was interpreted by the scores of the Medical Mistrust Multiformat Scale, the Dimensions of Anger Reactions, and the Cynical Distrust Scale. Medical mistrust mediated the effect of cynical distrust on vaccination hesitancy and anger moderated the indirect relationships between cynical distrust and vaccination hesitancy via medical mistrust.

## Figures and Tables

**Table 1:** Descriptive statistics of participants.

Participants		Age	Work Experience (in Years)	COVID-19 Vaccine Hesitancy	H_1_N_1_ Vaccine Hesitancy	Medical Mistrust Multiformat Scale	Cynical Distrust Scale	Dimensions of Anger Reactions	Total Vaccine Hesitancy
Men N = 66	Mean	45.53	19.26	3.36	2.48	19.97	22.58 **	10.35 *	5.85
	S. D	10.96	11.89	1.58	1.56	5.65	6.53	3.18	2.84
Women N = 321	Mean	43.04	17.78	3.62	2.72	20.73	24.96 **	11.48 *	6.33
	S. D	10.99	12.09	1.27	1.47	5.43	6.10	3.96	2.42
Total N = 387	Mean	43.46	18.03	3.58 **	2.68 **	20.60	24.55	11.29	6.25
	S. D	11.011	12.05	1.33	1.49	5.47	6.23	3.86	2.50

* *t*-test *p* > 0.05; ** *t*-test *p* > 0.01.

**Table 2:** Frequencies and percentages of vaccine hesitancy.

	In the Next Flu Shot (H_1_N_1_) Recommendation, Would You Get Vaccinated?		If a New Shot Dose Were Recommended as an Adjunct to the Current Vaccination Schedule for COVID-19, Would You Get Vaccinated?	
	Frequency	Percent	Frequency	Percent
I would definitely get vaccinated	125	32.3%	38	9.8%
I would probably get vaccinated	68	17.6%	51	13.2%
I’m not sure	68	17.6%	77	19.9%
I probably wouldn’t get vaccinated	59	15.2%	92	23.8%
I definitely wouldn’t get vaccinated	67	17.3%	129	33.3%
Total	387	100%	387	100%

**Table 3:** Correlations among age, work experience (in years), vaccine hesitancy, medical mistrust, and cynical distrust.

Pearson Correlation N = 387		Age	Work Experience (in Years)	COVID-19 Vaccine Hesitancy	H_1_N_1_ Vaccine Hesitancy	MMMS	CDS	DAR
Work experience (in years)	*r*	0.878 **						
COVID-19 vaccine hesitancy	*r*	−0.035	−0.020					
H_1_N_1_ vaccine hesitancy	*r*	−0.030	−0.023	0.571 **				
Medical Mistrust Multiformat Scale (MMMS)	*r*	−0.179 **	−0.125 *	0.249 **	0.180 **			
Cynical Distrust Scale (CDS)	*r*	−0.140 **	−0.110 *	0.203 **	0.207 **	0.408 **		
Dimensions of Anger Reactions (DAR)	*r*	−0.063	−0.051	0.197 **	0.208 **	0.384 **	0.453 **	
Total Vaccine Hesitancy	*r*	−0.036	−0.024	0.872 **	0.899 **	0.240 **	0.231 **	0.229 **

* Pearson Correlations *p* < 0.05, ** Pearson Correlations *p* < 0.01.

**Table 4:** Stepwise multiple regression (only statistically significant variables are included).

Dependent Variable: Total Vaccine Hesitancy	R Square	R Square Change	Beta	*t*	*p*	VIF	Durbin–Watson
Medical Mistrust Multiformat Scale	0.058	0.058	0.146	2.651	0.01 *	1.27	1.957
Dimensions of Anger Reactions	0.079	0.022	0.119	2.114	0.05 **	1.34	
Cynical Distrust Scale	0.090	0.010	0.118	2.065	0.05 **	1.37	

Notes: Beta = standardized regression coefficient; correlations are statistically significant at the * *p* < 0.01 level or ** *p* < 0.05.

**Table 5:** Mediation analysis of Medical Mistrust Multiformat Scale (MMMS) on Cynical Distrust Scale (CDS)—total vaccine hesitancy (TVH) relationship.

Variable	b	SE	*t*	*p*	95% Confidence Interval	
					LLCI	ULCI
CDS → MMMS	0.3408	0.0409	8.3313	0.0000	0.2603	0.4212
CDS → TVH	0.0924	0.0201	4.5889	0.0000	0.0528	0.1320
CDS → MMMS → TVH	0.0810	0.0248	3.2594	0.0012	0.0321	0.1298
Effects						
Direct	0.0648	0.0216	2.9983	0.0029	0.0223	0.1073
Indirect *	0.0276	0.0093	2.9677		0.0097	0.0470
Total	0.0924	0.0201	4.5889	0.0000	0.0528	0.1320

* Based on 5000 bootstrap samples. Abbreviations: CDS, Cynical Distrust Scale; MMMS, Medical Mistrust Multiformat Scale; TVH, total vaccine hesitancy. Note: Work experience and age were included in the analysis as covariate variables. They are not shown in the table as they did not give significant statistical results (*p* > 0.05).

**Table 6:** Moderated mediation analysis of the effect of Dimensions of Anger Reactions (DAR) on the association between Cynical Distrust Scale (CDS) and total vaccine hesitancy (TVH) through the Medical Mistrust Multiformat Scale.

Direct Relationships						
Variable	b	SE	*t*	*p*	95% Confidence Interval	
					LLCI	ULCI
CDS → MMMS	0.5875	0.1126	5.2155	0.0000	0.3660	0.8090
CDS → TVH	0.0642	0.0215	2.9867	0.0030	0.0219	0.1065
MMMS → TVH	0.0798	0.0245	3.2544	0.0012	0.0316	0.1280
CDS*DAR → MMMS	−0.0318	0.0100	−3.1800	0.0016	−0.0515	-0.0121
**Indirect Relationships**						
**Effects**						
Direct	0.0642	0.0215	2.9867	0.0030	0.0219	0.1065
Indirect	0.0182	0.0067	2.7164		0.0064	0.0322
**Probing Moderated Indirect Relationships**						
Low Level of DAR	0.0280	0.0097	2.8866		0.0101	0.0479
High Level of DAR	0.0084	0.0058	1.4482		−0.0017	0.0210
Index of Moderated Mediation	−0.0025	0.0011	2.2727		-0.0051	−0.0006

## Data Availability

The data that support the findings of this study are available from the corresponding author, A.T., upon reasonable request.
